# Adjusting vector surveillance for human behaviors reveals *Anopheles funestus* drove a resurgence in malaria despite IRS with clothianidin in Uganda

**DOI:** 10.1038/s41598-025-00623-0

**Published:** 2025-05-22

**Authors:** Paul Krezanoski, Alex Musiime, Ambrose Oruni, Max McClure, Patrick Kyagamba, Geoffrey Otto, James Adiga, Odol Wilfred, Moses Semakula, Jackson Asiimwe Rwatooro, Kilama Maxwell, Neil F. Lobo, Emmanuel Arinaitwe, Joaniter I. Nankabirwa, Moses Kamya, Grant Dorsey, Edward K. Thomsen

**Affiliations:** 1 University of California, San Francisco, CA USA; 2 National Malaria Control Division, Ministry of Health, Kampala, Uganda; 3 Liverpool School of Tropical Medicine, Liverpool, UK; 4 Infectious Diseases Research Collaboration, Kampala, Uganda; 5 University of Notre Dame, Notre Dame, IN USA; 6 Makerere University College of Health Sciences, Kampala, Uganda

**Keywords:** Epidemiology, Malaria

## Abstract

After remarkable success following the implementation of indoor residual spraying (IRS) and repeated rounds of universal distribution of insecticidal treated nets in Tororo District, eastern Uganda, a switch to clothianidin-based IRS in March 2020 was associated with a resurgence of malaria transmission. A previous study suggested *Anopheles funestus* may be driving the resurgence. This study was undertaken to assess the role of *An. funestus* in the resurgence and improve our understanding of how human-vector interaction affects malaria transmission in settings with extensive vector control. Using human landing catches and human behavioral observations, we found *An. funestus* infective biting, calculated from human-behavior adjusted biting rates and species-specific sporozoite rates, was 4.3 (95% Confidence Interval [CI]: 1.81 to 10.33) times higher after multiple rounds of clothianidin-based IRS when transmission was high and then dropped off markedly with a switch back to the organophosphate Actellic in March 2023. This finding was bolstered by a causal analysis showing a link between clothianidin-based IRS and 8.6 (95% CI: 2.0 to 37.0) times higher human-behavior adjusted human biting rates due to *An. funestus*. These findings highlight the importance of integrating monitoring of human-vector interaction and vector bionomics when introducing or evaluating changes in vector control interventions.

## Introduction

Indoor residual spraying (IRS) has shown remarkable success in controlling malaria transmission in many sub-Saharan African settings^1,2^. In the Tororo District of eastern Uganda, between 2014 and 2019, sustained IRS combined with two universal distributions of pyrethroid insecticidal treated nets (ITNs) was associated with a 60-fold decrease in the incidence of symptomatic malaria among children aged 0.5–10 years^3^. Despite another mass distribution of pyrethroid ITNs in 2020, a resurgence of malaria occurred coincident with a change to clothianidin-based IRS in March 2020. A previous study reported that the relative abundance of *Anopheles funestus* collected from indoor CDC light traps increased during this time, suggesting this vector may have been a key driver of the resurgence^4^. While clothianidin resistance has been reported among *An. gambiae sensu lato* vectors in Western Africa^5,6^, only recently has evidence shown that *An. funestus* may have moderate resistance to clothianidin in Uganda^7^. This study was therefore undertaken to obtain a clearer picture of the human-vector exposure patterns during this time period.

**Fig. 1:**
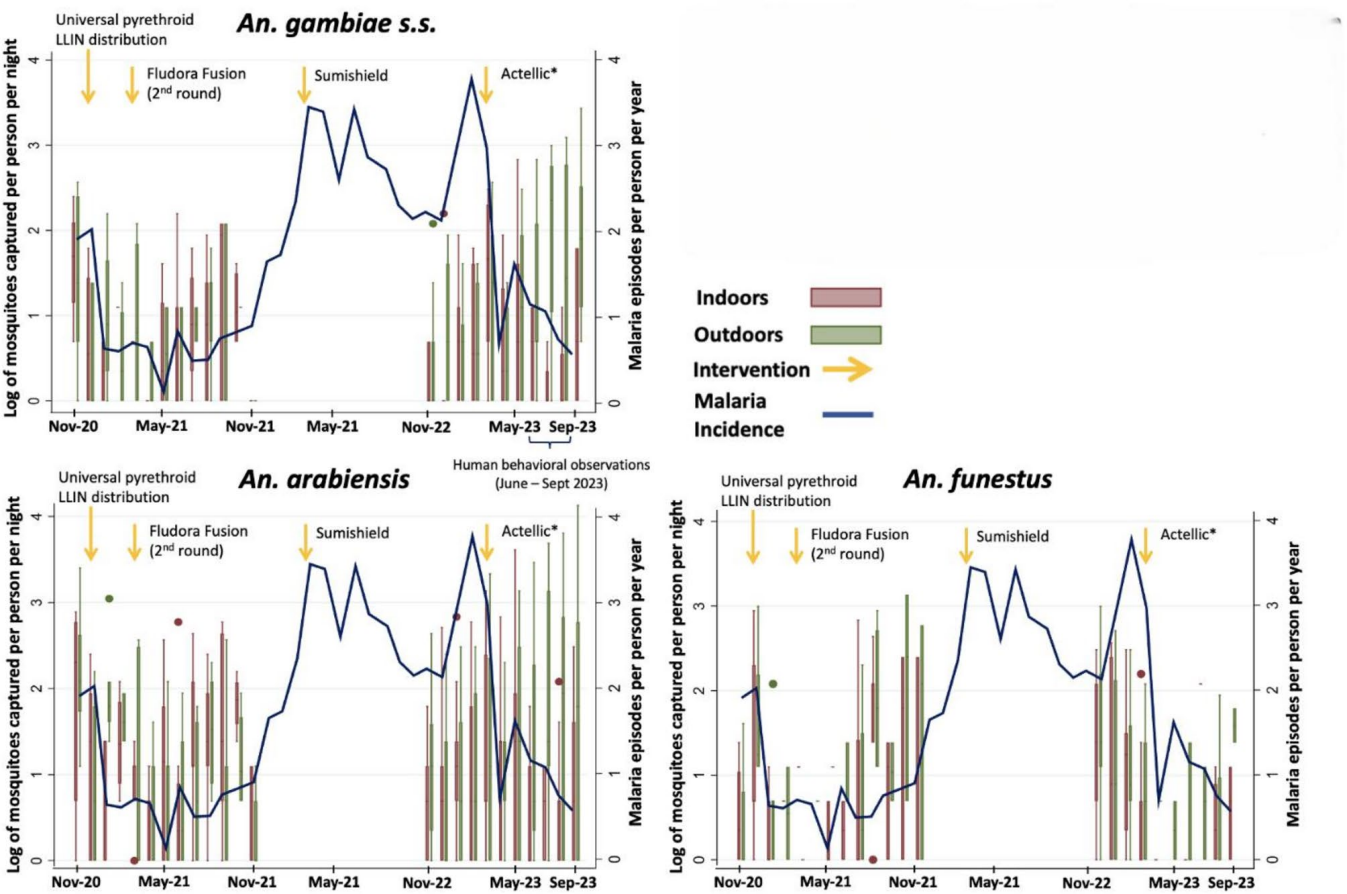
Box plots from indoor and outdoor human landing collections and malaria incidence from household-based cohort in eastern Uganda. Arrows denote vector control interventions. Separate panels indicate *Anopheles* species on same log scale.

Tororo is historically a high malaria transmission district with an estimated entomological inoculation rate (EIR) of 310 infective bites per person per year in 2011 − 12^8^. In November 2013, universal distribution of pyrethroid ITNs was conducted, and similar campaigns with pyrethroid ITNs were repeated in May 2017 and June 2020. IRS with a carbamate-based formulation, active ingredient bendiocarb, was introduced for the first time in December 2014–January 2015, with additional rounds administered in June–July 2015 and November–December 2015. In June–July 2016, the IRS formulation was changed to Actellic which contains an organophosphate, primiphos-methyl, as the active ingredient. There were repeated rounds of Actellic in June–July 2017, June–July 2018, and March–April 2019. In March-April 2020, the IRS formulation was changed to Fludora Fusion (a combination of clothianidin and deltamethrin) with a repeated round in March 2021 (Fig. 1). In March 2022, a formulation with clothianidin alone, Sumishield, was used. In February-March 2023, a decision was made to shift back to IRS with Actellic in response to the resurgence of malaria, although some households, including three from this study, received another round with Sumishield to use up remaining stocks.

This study was undertaken to further explore how *An. funestus* may have contributed to the malaria resurgence in Tororo District and improve our understanding of how entomological and human factors affect malaria transmission in a setting with extensive vector control. We performed entomological surveillance in two phases, from November 2020 to November 2021 (Phase 1) and from November 2022 to September 2023 (Phase 2), and calculated three metrics of human-vector interaction: (1) human biting rates derived directly from HLCs (HBRs), (2) human-behavior adjusted HBRs which integrate HBOs (aHBRs) and (3) adjusted infective biting rates representing an integrated measure of species-specific aHBRs and species-specific sporozoite rates. By comparing these metrics over the course of changing IRS formulations we sought to characterize human and vector factors associated with the resurgence in malaria transmission experienced in Tororo from 2020 to 2023 and the marked decline in transmission after the IRS formulation was switched back to the organophosphate, Actellic.

## Results

### Characteristics of household cohorts

Household demographic data was only gathered from the 12 households that took part in the HBOs in Phase 2. At that time, households had a mean number of 4.0 inhabitants (range: 1 to 8), 56% were female and median age was 13 years (interquartile range: 7 to 35). These households had a mean of 1.2 bednets (range: 0 to 2) and 1.6 sleeping rooms (range: 1 to 4). These characteristics were comparable to those of the 80 households in the clinical cohort which are detailed elsewhere^4^.

### Entomological and clinical indicators

The HLCs captured 1023 *An. gambiae s.l.* and 357 *An. funestus sensu lato* mosquitoes in Phase 1 and 2461 *An. gambiae s.l.* and 507 *An. funestus s.l.* in Phase 2. Of the 403 *An. gambiae s.l.* mosquitoes tested by PCR from the first half of Phase 2, 73.5% were *An. arabiensis* and 26.5% were *An. gambiae sensu stricto* (henceforth referred to as *An. gambiae*). There was no significant difference between the proportion of *An. arabiensis* collected indoors and outdoors (data not shown). All 126 *An. funestus s.l.* tested by PCR during Phase 2 were determined to be *An. funestus sensu stricto* (henceforth referred to as *An. funestus*). Figure 1 summarizes female *Anopheles* mosquitoes captured by species overlaid with clinical malaria incidence (as measured in the clinical cohort) and vector control interventions. Malaria incidence decreased from November 2020 through February 2021 coinciding with declining abundance of all three *Anopheles* species. During this time period, the HBR for *An. funestus* was 1.7 mosquitoes per person per night (20.5% of the total). From March to November 2021, following the second round of IRS with Fludora Fusion, abundances of all species increased slightly, though the *An. funestus* HBR was similar at 1.5 per person per night (29.2% of total). When collections resumed in November 2022, abundances remained high, but now the HBR from *An. funestus* had increased to 2.3 (43.0% of the total). Following IRS with Actellic in February to March 2023, coinciding with a dramatic reduction in incidence, the relative abundances of *An. arabiensis* and *An. gambiae* were much greater than *An. funestus* whose HBR had fallen to 0.2 per night (3.4% of the total) (Fig. 1).

### Human and vector behaviors

**Fig. 2:**
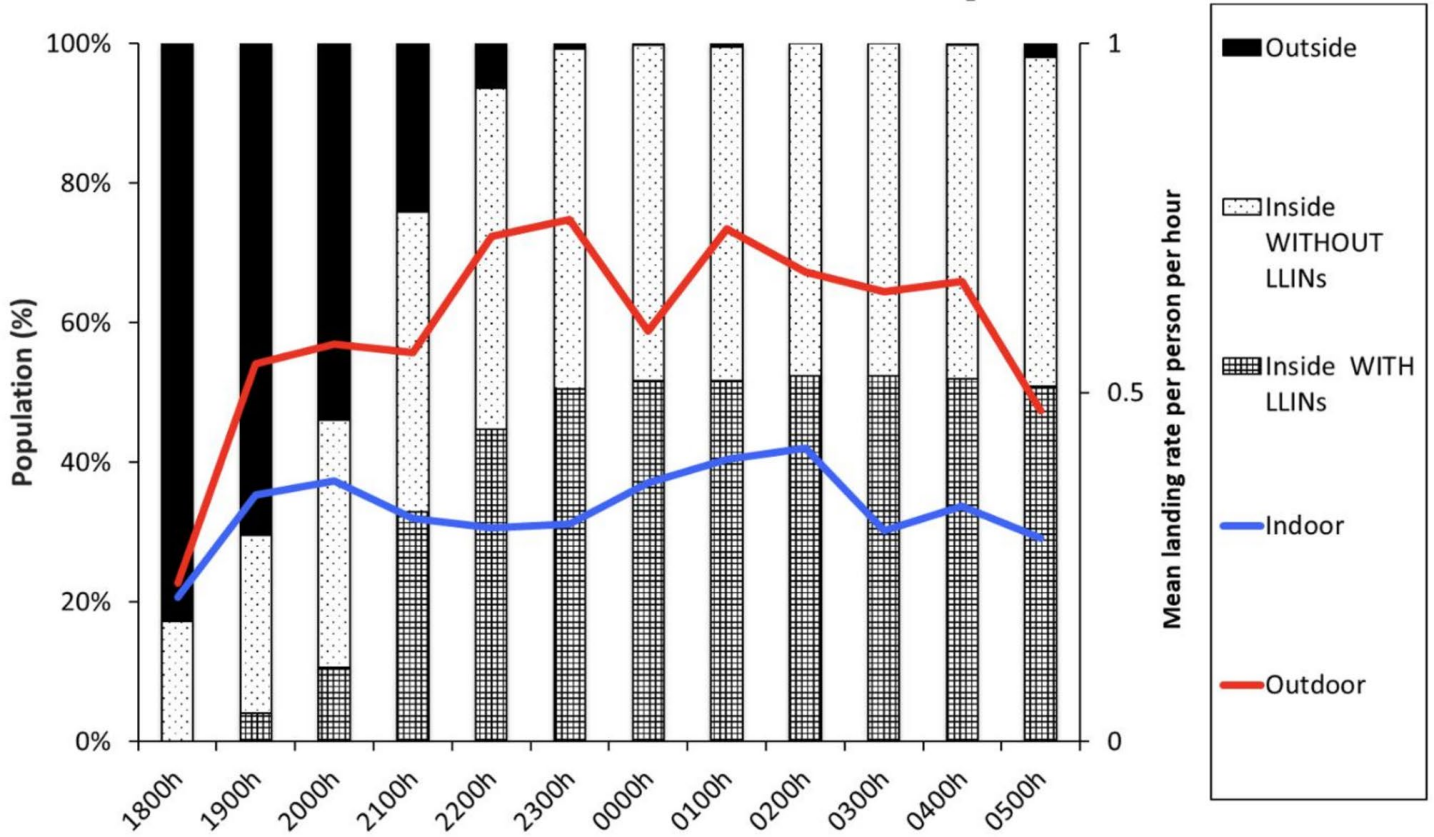
Proportion of people observed by activity and *Anopheles* indoor and outdoor biting rates by night hour in eastern Uganda. Data from biweekly human landing collections over all time periods and human behavioral observations in 12 households from June to September 2023.

During Phase 2, HBOs were performed on the same nights and in the same households as the HLCs from June 2023 until September 2023. The proportion of individuals observed to be outside decreased from 82% between 1800 and 1900 h to 50% between 2000 and 2100 h to nearly 0% by 2300 h (Fig. 2). By 2300 h, observed ITN use had reached its maximum at around 50%, with the other 50% of the population inside, presumably sleeping, without an ITN. Hourly all-species HBRs were higher outdoors than indoors, with a mean of 0.62 mosquitoes per person per hour (ppph) outdoors (95% CI: 0.57 to 0.68) compared to 0.27 ppph (95% CI: 0.24 to 0.30) indoors (Fig. 2).

**Fig. 3:**
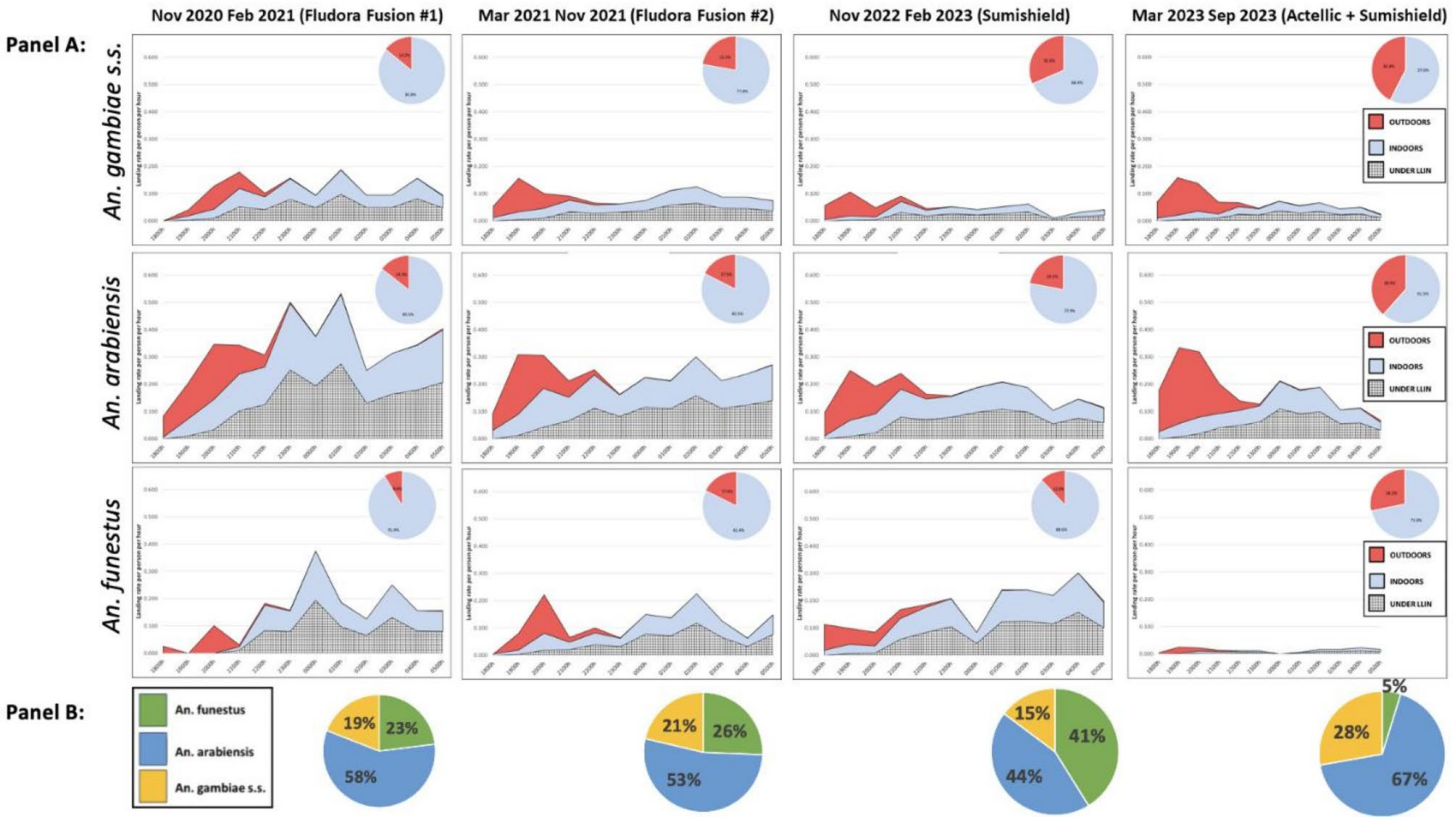
Human behavior-adjusted landing rates. **Panel A**: Human behavior-adjusted landing rates per person by hour and exposure compartment by species. **Panel B**: Proportion of adjusted landing by species.

Human exposure to mosquito bites Human-behavior adjusted HBRs, aHBRs, were calculated as an estimate of hourly exposure by accounting for observed temporal and spatial human presence (including ITN usage behaviors). The proportion of exposure to each vector species occurring outdoors increased over time, with a pronounced increase after IRS with Actellic (Fig. 3: Panel A). Exposure to *An. gambiae*, even during the high malaria incidence period, was markedly lower than the other two species. After Actellic was reintroduced exposure to *An. funestus* dropped dramatically and exposure to *An. arabiensis* and *An. gambiae* remained consistent, but shifted to earlier hours of the evening and outdoors. Compared to Phase 1, the proportion of exposure due to *An. funestus* was higher during the resurgence and then dropped precipitously after the re-introduction of Actellic (Fig. 3: Panel B).

Estimates of overall nightly exposure derived from the aHBRs were lower than raw HBRs, but *An. funestus* made up essentially the same proportions in both. In terms of exposure to all *Anopheles* species, there were no significant differences in aHBRs comparing the first to any other time period after accounting for human behaviors and adjusting for season, proximity to the border and clustering at the household level. (Table 1). When evaluating aHBRs by species, there was a marked decrease in the *An. funestus* aHBR after the switch to Actellic compared to the first time period with a RR 0.18 (95% CI: 0.05 to 0.69), but no significant increase during the period with high transmission (Table 2). The estimated aHBR from *An. arabiensis* was more than 40% lower during the malaria resurgence in the third time period compared to the first (RR: 0.59, 95% CI: 0.37 to 0.94). There were no significant differences in aHBRs from *An. gambiae* across any of the time periods (joint significance p-value = 0.486).

**Table 1:** Human biting and infective biting rates from all *Anopheles* species adjusted for host availability from observed human behaviors.

	Human-behavior adjusted HBRs*				Infective biting rate***			
	Raw	Regression-adjusted**			Raw	Regression-adjusted		
	Rate (95% CI)	Rate (95% CI)	Rate ratio (95% CI)	*p*-value	Rate (95% CI)	Rate (95% CI)	Rate ratio (95% CI)	*p*-value
Nov 2020 to Feb 2021	4.0 (2.2 to 5.8)	3.6 (1.3 to 5.9)	Ref		0.05 (0.03 to 0.07)	0.05 (0.02 to 0.06)	Ref	
Mar 2021 to Nov 2021	3.2 (2.3 to 4.2)	2.9 (1.2 to 4.7)	0.81 (0.52 to 1.23)	0.316	0.04 (0.03 to 0.05)	0.03 (0.01 to 0.06)	0.85 (0.57 to 1.26)	0.407
Nov 2022 to Feb 2023	3.0 (2.1 to 3.8)	2.9 (1.6 to 4.3)	0.82 (0.52 to 1.3)	0.391	0.07 (0.05 to 0.09)	0.08 (0.04 to 0.11)	1.92 (1.25 to 2.95)	0.003
Mar 2023 to Sep 2023	2.3 (1.6 to 2.9)	2.5 (0.8 to 4.1)	0.69 (0.35 to 1.35)	0.274	0.02 (0.01 to 0.02)	0.02 (0.01 to 0.03)	0.48 (0.25 to 0.92)	0.026

*Human-behavior adjusted HBRs calculated as the sum over the night of vector exposure determined per hour when individuals are outdoors or indoors without bednet use.

**Marginal estimates and rate ratios from negative binomial regression models accounting for clustering at household level and adjusted for season and proximity to border.

***Infective biting rates based on species-specific nightly landing rates accounting for human behaviors and species-specific sporozoite rates from CDC LTs.

**Table 2:** Human biting and infective biting rates adjusted for host availability from observed human behaviors by species.

	Human-behavior adjusted HBRs*						Infective biting rates***					
	An. funestus		An. arabiensis		An. gambiae s.s.		An. funestus		An. arabiensis		An. gambiae s.s.	
	Rate ratio (95% CI)	*p*-value	Rate ratio (95% CI)	*p*-value	Rate ratio (95% CI)	*p*-value	Rate ratio (95% CI)**	*p*-value	Rate ratio (95% CI)	*p*-value	Rate ratio (95% CI)	*p*-value
Nov 2020 to Feb 2021	Ref		Ref		Ref		Ref		Ref		Ref	
Mar 2021 to Nov 2021	0.82 (0.55 to 1.22)	0.322	0.74 (0.44 to 1.24)	0.250	0.90 (0.37 to 2.20)	0.820	0.97 (0.64 to 1.48)	0.887	0.63 (0.39 to 1.04)	0.071	1.439 (0.59 to 3.26)	0.449
Nov 2022 to Feb 2023	1.96 (0.82 to 4.67)	0.131	0.59 (0.37 to 0.94)	0.025	0.60 (0.25 to 1.44)	0.250	4.33 (1.81 to 10.33)	0.001	1.02 (0.67 to 1.57)	0.923	1.41 (0.57 to 3.47)	0.458
Mar 2023 to Sep 2023	0.18 (0.05 to 0.69)	0.013	0.71 (0.36 to 1.42)	0.330	0.88 (0.29 o 2.67)	0.819	0.19 (0.05 to 0.71)	0.014	0.29 (0.15 to 0.56)	< 0.001	1.32 (0.45 to 3.91)	0.611

* Human-behavior adjusted HBRs calculated as the sum over the night of vector exposure determined per hour when individuals are outdoors or indoors without bednet use.

** Estimated *An. gambiae s.l.* sub-complex designation based off PCR prevalence from random sample .

***Rate ratios from negative binomial regression models accounting for clustering at household level and adjusted for season and proximity to border.

†Infective biting rates based on species-specific nightly landing rates accounting for human behaviors and species-specific sporozoite rates from CDC LTs.

### Vector infectivity rates

Sporozoite rates in mosquitoes from indoor CDC light traps in the clinical cohort were consistently higher in *An. funestus* than *An. gambiae s.l.* (Table 3). Sporozoite rates across all species were highest between November 2022 and February 2023, when *An. funestus* positivity rate was 3.2% (95% CI: 2.3–4.0%), *An. arabiensis* was 1.8% (1.4 to 2.2%) and *An. gambiae* was 2.2% (1.8–2.7%). When Actellic was reintroduced in most of the district in March 2023, sporozoite rates dropped in all species.

**Table 3:** Sporozoite rates by species from biweekly indoor CDC light traps in clinical cohort households.

	An. funestus		An. gambiae s.l.		An. gambiae complex*	
					An. arabiensis	An. gambiae s.s.
	Positive/tested	Sporozoite rate (95% CI)	Positive/tested	Sporozoite rate (95% CI)	Sporozoite rate (95% CI)	Sporozoite rate (95% CI)
Nov 2020 to Feb 2021	32/2329	1.37% (0.90–1.85%)	70/6765	1.03% (0.80–1.28%)	1.13% (0.74–1.51%)	0.97% (0.65–1.28%)
Mar 2021 to Nov 2021	123/7609	1.62% (1.33–1.90%)	153/12,793	1.20% (1.01–1.38%)	0.95% (0.73–1.17%)	1.51% (1.20–1.82%)
Nov 2022 to Feb 2023	54/1704	3.17% (2.34–4.00%)	24/1215	1.98% (1.19–2.76%)	1.77% (1.36–2.18%)	2.23% (1.77 to 2.69%)
Mar 2023 to Sep 2023	36/2433	1.48% (1.00–1.96%)	27/3210	0.84% (0.53–1.16%)	0.42% (0.13–0.72%)	1.48% (0.83–2.12%)

*Estimated based on prevalence using PCR testing on random sub-sample of total Anopheles gambiae s.l.

### Exposure to infective bites

**Fig. 4:**
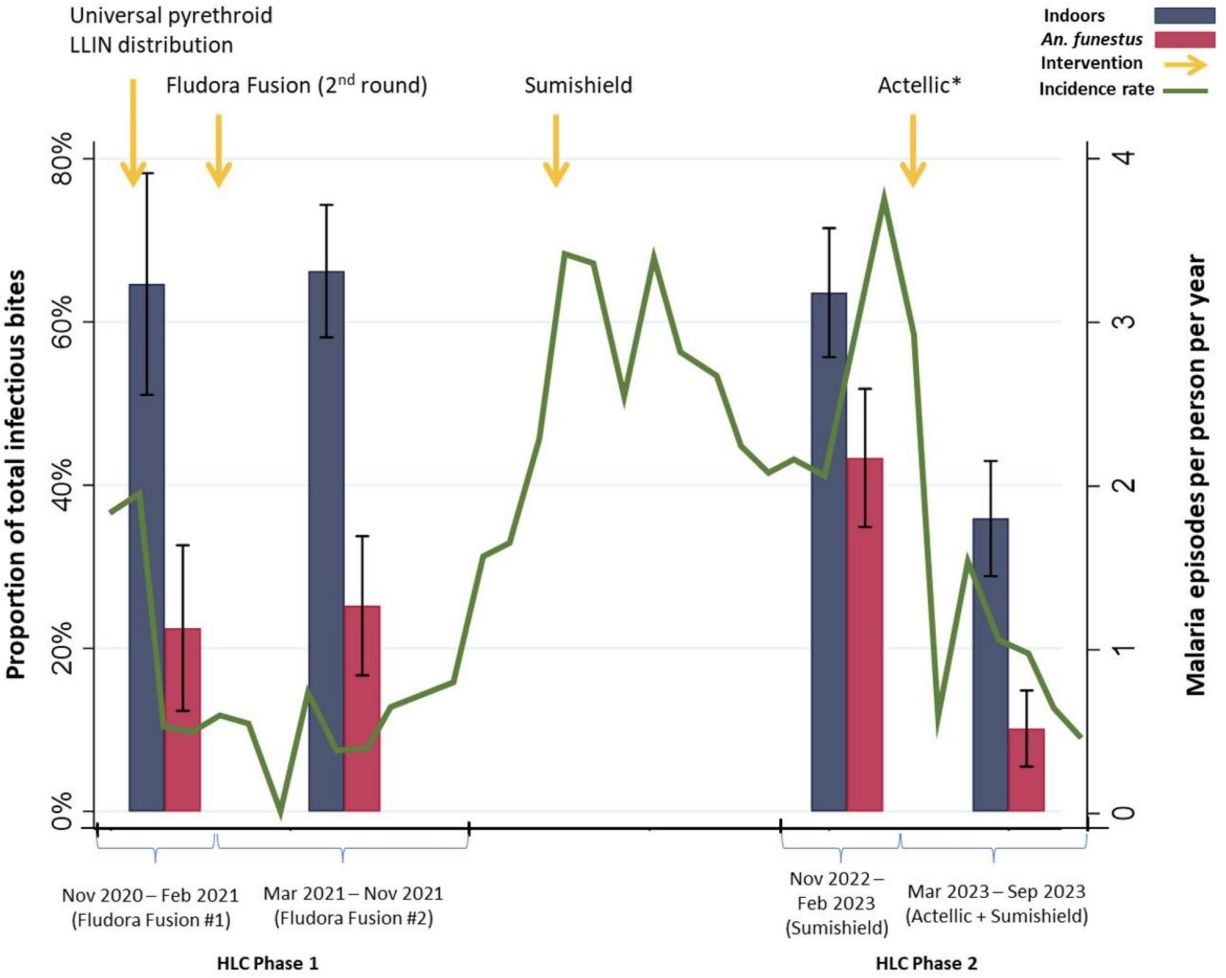
The proportion of infective biting from all *Anopheles* species occurring indoors and the proportion of infective biting (indoor plus outdoor) attributable to *An. funestus* over time.

We estimated nightly adjusted infective biting rates that considered both species-specific sporozoite rates and species-specific HBRs adjusted for human behaviors. This measure, not the same as entomological inoculation rate (EIR), incorporates both vector infectivity and human-behavior adjusted exposure to estimate mean individual exposure to infective bites. We found a nearly two times higher overall *Anopheles* infective biting rate during the resurgence (November 2022-February 2023) compared to the first time period (November 2020-February 2021) with a risk ratio (RR) of 1.92 (95% CI: 1.25 to 2.95). The proportion of infective biting occurring indoors was similar across the first three time periods (Fig. 4), including during the malaria resurgence when 63.6% (95% CI: 55.6–71.1%) of the estimated 0.08 (0.04 to 0.11) infective bites per person per night occurred indoors. The magnitude of infective biting dropped after the introduction of Actellic in March 2023 to 0.02 (95% CI: 0.01 to 0.03) bites per person per night, with 35.9% (95% CI: 28.9 to 43.0%) of these occurring indoors. Compared to the first time period, infective biting rates were more than halved after the switch back to Actellic with a RR of 0.48 (95% CI: 0.25 to 0.92) (Table 1). These observations track malaria incidence trends over those time periods (Fig. 1).

Compared to the first time period, exposure to infective bites from *An. funestus* was 4.3 (95% CI: 1.8 to 10.3) times higher during the malaria resurgence. These then decreased by 80% after Actellic was introduced and malaria incidence fell (RR: 0.2, 95% CI: 0.1 to 0.7) (Table 2). Adjusted infective biting due to *An. arabiensis* also declined markedly after Actellic was reintroduced compared to the first time period (RR: 0.3; 95% CI: 0.2 to 0. 6), despite aHBR not being statistically different (*p* = 0.330). Exposure to infective bites from *An. gambiae* was not significantly different across any of the time periods (*p* = 0.850). The proportion of infective biting due to *An. funestus*, however, showed a marked increase during the time of the malaria resurgence to 43.3% (95% CI: 34.9–51.8%). This was significantly higher than either the first (*p* = 0.011) or second time (*p* = 0.003) periods. Furthermore, after the introduction of Actellic, the proportion of overall infective biting due to *An. funestus* dropped to 10.2% (95% CI: 5.5–14.8%), which was the lowest of all time periods (*p* = 0.017). Of note, the proportions of exposure attributable to *An. funestus* across all three metrics, the raw HBR, aHBR and the infective biting rate, were essentially the same in each time period.

### Causal analysis of IRS product on *An. funestus* adjusted landing rates

We performed a difference-in-difference analysis restricted to eight households that were all near the border with Busia District where IRS has never been implemented. This analysis took advantage of the fact that three of these households received a second round of the clothianidin-based Sumishield after February-March 2023, while the other five households received the organophosphate Actellic like most households in the rest of the Tororo District. This analysis showed that, after controlling for household-level characteristics, secular trends and unobserved time-invariant factors, there was no significant difference between households that received Sumishield or Actellic in nightly aHBRs when combining all *Anopheles* species (RR: 1.3; 95% CI: 0.5 to 3.4) (Table 4). However, households that received Sumishield compared to Actellic had 8.6 times higher *An. funestus*-specific aHBRs (95% CI: 2.0 to 37.0).

**Table 4:** Difference-in-difference analysis of households receiving actellic compared to households continuing to receive Sumishield in Feb-Mar 2023 IRS round.

			All Anopheles species human-behavior adjusted HBRs*				An. funestus human-behavior adjusted HBRs			
Intervention	Households**	Observations	Pre-Mar 2023 (95% CI)	Post-Mar 2023 (95% CI)	Rate ratio (95% CI)***	*p*-value	Pre-Mar 2023	Post-Mar 2023	Rate ratio (95% CI)	*p*-value
Sumishield to Actellic	5	185	5.2 (1.3 to 9.0)	2.1 (0.1 to 4.2)	Ref		1.2 (0.2 to 2.3)	0.1 (0.0 to 0.1)	Ref	
Continue Sumishield	3	83	3.4 (0.2 to 6.7)	1.8 (0.5 to 3.2)	1.3 (0.5 to 3.4)	0.614	0.9 (0.0 to 2.0)	0.5 (0.0 to 1.0)	8.6 (2.0 to 37.0)	0.004

*Human-behavior adjusted HBRs calculated as the sum of vector exposure per hour when individuals are outdoors or indoors without bednet use.

**Analysis restricted to the 8 households clustered near the border to control for geographic differences.

***Rate ratios from negative binomial regression models over entire observation period, adjusted for seasonality and accounting for clustering at household level.

## Discussion

Indoor residual spraying (IRS) has been associated with notable success in controlling malaria in sub-Saharan Africa, including Uganda^2,3^. Despite continued use of IRS over seven years, and regular ITN distribution, Tororo District experienced an unexpected resurgence in malaria from 2021 to 2023 that was temporally correlated with a change in formulation to clothianidin-based insecticide products Fludora Fusion and Sumishield^4^. This study used detailed measures of human and vector behavior to quantify human-vector interaction and better understand what factors were associated with the resurgence in malaria. The findings strongly suggest *An. funestus* was driving malaria incidence and hence transmission in the human population. During the resurgence, we found that the infective biting rate from *An. funestus* was 4.3 times higher than a comparable time period two years previously, while both human-behavior adjusted HBRs and infective biting rates from *An. arabiensis* and *An. gambiae* were either stable or lower than baseline values. In addition, the re-institution of Actellic in February-March 2023 was temporally correlated with an 82% decrease in aHBRs and 81% decrease in infective biting rates from *An. funestus* that coincided with the drop in malaria transmission from 0.08 to 0.02 bites per person per night. While the proportion of infective biting occurring indoors was stable through the surge, the proportion of infective biting due to *An. funestus* was significantly higher during the resurgence than any other time period, and then was significantly lower once Actellic was reintroduced. Finally, in a difference-in-difference analysis restricted to geographically proximal households, we found a causal association between receiving clothianidin-based IRS in the final round and 8.6 times higher aHBRs due to *An. funestus* compared to households that received Actellic.

This study adds to evidence supporting a causal link between the change of IRS formulation from the organophosphate Actellic to clothianidin-based formulations in this region of Uganda and a resurgence in malaria due to increased exposure to *An. funestus*. A similar resurgence in malaria cases has been reported in four other districts of Uganda^9^. Recently, in a study of our clinical cohort in Tororo, we found evidence that *An. funestus* showed high tolerance to clothianidin but was fully susceptible to primiphos-methyl, the active ingredient in Actellic^4^. The latter study also found that *An. funestus* was the predominant vector captured using indoor CDC-light traps during the malaria resurgence. In contrast, using HLCs, the raw HBRs from *An. funestus* in our study made up 20–29% of the total HBR pre-resurgence and then reached 43% during the peak resurgence before dropping off precipitously. While there were no significant differences between the HBRs or aHBRs during the resurgence and those of earlier time periods, comparing adjusted infective biting rates, which incorporate both species-specific aHBRs and species-specific sporozoite rates, showed a strong and plausible temporal correlation between *An. funestus* infective biting rates and the malaria resurgence. This result suggests that (1) infective biting due to *An. funestus* reached a relative tipping point during the resurgence period that was associated with a significant increase in malaria transmission and (2) integrated measures of human-vector interaction and vector bionomics utilizing species-specific behaviors and sporozoite rates are crucial tools for uncovering drivers of malaria transmission.

Multiple studies of clothianidin-based IRS formulations have shown good effects against both *An. gambiae s.l.* and *An. funestus* under experimental conditions^10,11^ and in field trials in Kenya, Benin and Cote d’Ivoire^12–15^. Some studies have shown reduced susceptibility to Sumishield in studies of different building materials^5^ and resistance to clothianidin in *An. gambiae s.l.* populations in Cameroon^6^. A study in Tanzania similarly found that *An. funestus* mosquitoes showed high degrees of resistance to multiple insecticide classes (though notably clothianidin was not tested) and although they made up only 19% of *Anopheles* species collected via CDC LTs and indoor Prokopak aspirations, *An. funestus* accounted for 73% of the total entomological inoculation rate^15^. The only other study of the clinical impacts of clothianidin-based IRS also showed that Fludora Fusion had no impact in Northern Zambia where *An. funestus* is the primary vector^16^. Finally, a recent study from Uganda in a district adjacent to where this study was performed showed moderate resistance to clothianidin among *An. funestus*^7^, providing additional evidence for this phenomenon.

Combining observations of human behaviors including time spent outdoors and under ITNs, we showed that the proportion of exposure to biting mosquitoes occurring indoors decreased with subsequent rounds of clothianidin-based IRS formulations for *An. arabiensis and An. gambiae*, but remained 80–90% for *An. funestus* (Fig. 3: Panel A). These findings are similar to a recent study from Zambia that found persistently high levels of exposure to infective *An. funestus* bites, even in populations of children with higher ITN use (> 70%) than in our study^17^. After the introduction of Actellic, absolute exposure to *An. funestus* nearly disappeared, and the proportion of exposure occurring indoors dropped to 70%, consistent with a more effective lethal effect on *An. funestus* populations by Actellic compared to clothianidin-based IRS^4^. Furthermore, while the absolute exposure to *An. arabiensis* and *An. gambiae* remained essentially stable, exposure shifted markedly outdoors. Absent any other interventions besides application of Actellic IRS, this shift outdoors among *An. arabiensis* and *An. gambiae* appears to be most consistent with an effective intervention selecting for vectors preferring to bite outdoors during the hours of the night that individuals unprotected by ITNs might otherwise be susceptible. A high proportion of biting occurred outdoors after the re-introduction of Actellic, with most of the transmission during this time period driven by *An. arabiensis*. A transition towards *An. arabiensis* becoming the predominant malaria vector is similar to what we have observed previously with the initial introduction of Actellic in this region^18^.

One limitation of this study is that the lower number of *Anopheles* vectors captured with the HLCs was insufficient to provide meaningful estimates of sporozoite rates by species and collection location. As a result, we used sporozoite rates by species based on indoor CDC LT collections and were unable to differentiate between sporozoite positivity indoors versus outdoors. In addition, the clinical cohort from which we calculated malaria incidence, and from which sporozoite rates were derived, was performed in separate households than the HLCs. Nevertheless, the findings in this clinical cohort were also shown to track with malaria incidence estimated from health centers in Tororo during these time periods^4^, making it likely that the HLC households were experiencing similar malaria transmission. Estimates of the proportion of *An. gambiae s.l.* sub-species was based on relative proportions of indoor and outdoor *An. arabiensis* and *An. gambiae* by collection hour from the first half of Phase 2 due to labelling issues. This could have altered some conclusions comparing *An. arabiensis* to *An. gambiae* but would not affect conclusions related to *An. funestus*. HBOs were only performed from June to September 2023 and these data were used to generate exposure estimates across all time points. Human behaviors vary with seasons and we do not have data on human behaviors before June 2023 when transmission levels were higher, so it is possible that this could have biased exposure estimates. In addition to the surveillance teams, household residents were present during collection nights and this could have affected the yield from the indoor and outdoor HLCs. Vector abundance is also highly seasonal, so the time periods that were chosen for comparisons may have biased the results. The time periods were chosen to capture the effects during (1) the first 6 months after the spraying in February-March of each year and (2) the subsequent 6 months before the next round. In addition, in sensitivity analyses, there were no significant changes in the conclusions when using a different reference for regression comparisons (e.g. Mar 2021-Nov 2021 for comparing abundance in Mar 2023-Sept 2023) (data not shown).

## Conclusion

This region in Eastern Uganda had previously achieved successful malaria control with regular universal ITN distribution and continued IRS over 5 years before switching to clothianidin-based IRS in March 2020. Our findings shed light on how human and vector behaviors can contribute to the loss of control of malaria even after highly successful vector interventions. The integration of human behavioral observations with species-specific vector behaviors and sporozoite rates revealed *An. funestus* as the driver of the resurgence of malaria in this region. While indoor exposure remained stable before and during the surge, infective biting rates from *An. funestus* increased significantly during the resurgence. The re-introduction of Actellic in March 2023 was associated with a steep drop in malaria transmission when exposure shifted outdoors and *An. funestus* exposure dropped markedly, with evidence of a causal relationship between receiving clothianidin-based IRS and higher *An. funestus* aHBRs. These findings highlight the importance of integrating measures of human-vector interaction, vector bionomics and resistance testing for all relevant vector species before initiating, and during the early phases of, changes to vector control programs.

## Methods

### Human-vector interaction cohort: screening and procedures

Screening and enrollment of households has been described previously^19^. Briefly, in May 2020, households in two sub-counties in Tororo District were enumerated to provide a sampling frame. Human landing catches were carried out in two phases. From November 2020 to November 2021, 8 households were recruited for HLCs every 4 weeks for a total of 112 collection nights. In November 2022, in response to the unexpected malaria resurgence, HLCs were started again in 12 households every 2 weeks until September 2023 for a total of 276 collections nights. Phase 2 included 6 households from the first phase. Our protocol for HLCs was based on World Health Organization methods using one collector sitting indoors and one outdoors^20^. Each collector performed aspirations of landing mosquitoes for 50 min each hour from 6pm until 6am. Household members were present and performing their regular activities during the collection nights. In Phase 2, from June until September 2023, HBOs were also performed on the same nights in the same households as the HLCs. Following established methods^21^, these observations tracked the location of each individual in the household during the 10-minute break from HLCs each hour of the night from 6pm until 6am, recording whether each individual was (1) outdoors, (2) indoors without an ITN, or (3) under an ITN. No HLCs or HBOs were performed during the 11 months between phases.

### Clinical cohort

A separate cohort of 80 households was used to assess malaria incidence and sporozoite rates among malaria vectors in Tororo. This cohort was also chosen randomly from the same sampling frame from two sub-counties in the Tororo District as previously described^4^. Individuals in these households had access to free health care at a dedicated study clinic. The incidence of symptomatic malaria in the clinical cohort was defined as the number of incident cases of malaria (fever plus a positive thick blood smear by microscopy) per person per year. Episodes of malaria occurring within 10 days of a prior episode were not considered incident events. These households were also monitored with indoor CDC light traps every 4 weeks (Model 512; John W. Hock Company, Gainesville, Florida, USA). The CDC LTs were placed 1 m above the floor in all sleeping rooms from 7pm to 7am the following morning. Mosquitoes captured via CDC LT were used to calculate the species-specific sporozoite rates utilized in calculating infective biting rates for this study.

### Entomological lab methods

All female *Anopheles* from the HLCs and CDC light traps were enumerated and identified to the sibling species level based on morphological criteria according to established taxonomic keys^22^. From the HLCs, randomly selected mosquitoes identified to be a part of the *An. gambiae s.l.* complex underwent polymerase-chain reaction (PCR) analysis to distinguish between *An. gambiae* and *An. arabiensis*^23^. PCR testing was performed on all sporozoite positive *An. gambiae s.l.* and then randomly selected additional samples to achieve 20 total per collection period (4 weeks in Phase 1 and 2 weeks in Phase 2). During Phase 2 of the HLCs, a selection of *Anopheles* identified as *An. funestus s.l.* were tested via PCR to distinguish sub-complexes of the *An. funestus* species^24^. This analysis included all of the *An. funestus* samples that were sporozoite positive and a random selection of ~ 10% of the remaining samples. Sporozoite rates from the CDC light traps were assessed in up to 50 mosquitoes per collection using ELISA^27^ and up to 30 *An. gambiae s.l.* mosquitoes every 2 weeks were randomly selected to distinguish *An. gambiae* from *An. arabiensis* via PCR.

### Statistical analysis

All data were collected using standardized case record forms and double-entered using Microsoft Access (Microsoft Corporation, Redmond, Washington, USA). Analyses were performed using Stata, version 14 (Stata Corporation, College Station, Texas, USA). Given the timing of changes in IRS formulations and to allow comparisons of comparable seasons which are highly associated with vector density, the analyses were separated into four time periods, two each from each Phase: November 2020 to February 2021 (HLC Phase 1), March 2021 to November 2021 (HLC Phase 1), November 2022 to February 2023 (HLC Phase 2) and March 2023 to September 2023 (HLC Phase 2).

Insufficient numbers of sporozoite positive mosquitoes were captured via HLCs, so species-specific sporozoite rates for each time period from CDC light trap specimens were applied to both indoor and outdoor mosquitoes captured via the HLCs. For the CDC light traps, the number of *An. gambiae* and *An. arabiensis* collected were estimated by multiplying the observed proportions using the subset tested with species PCR by the total number of *An. gambiae s.l.* collected stratified by time period. For the HLCs, inadequate labelling led to an inability to identify indoor and outdoor mosquitoes by *An. gambiae s.l.* sub-species in Phase 1. Therefore, the relative proportions of indoor and outdoor *An. arabiensis* and *An. gambiae* by collection hour from the first half of Phase 2 (November 2022 to February 2023) were used to generate the proportions of the sub-species per hour for the other time periods.

Indoor and outdoor HBRs were defined as the number of female *Anopheles* mosquitoes collected per collector per hour. Human behavior-adjusted biting rates (aHBRs) were generated using HLCs and HBOs as in Monroe et al., 2020^26^. Individuals using an ITN were assumed to receive no exposure. Nightly infective biting rates were obtained by multiplying the aHBR by the sporozoite rate for that time period (and by species where applicable).

Comparisons of measures of aHBRs and infective biting rates by time period were made using negative binomial regression with HLC collections included as an offset and accounting for clustering at the household level. Co-variates included the season (January – June or July – December) and proximity to the border of the neighboring non-IRS Busia district (“near” or “away”). We used Wald tests to evaluate statistical differences between the marginal effects across different time periods. Comparisons of means were made using student’s t-test. To measure causal effects of different IRS formulations, a difference-in-difference analysis was performed. This analysis took advantage of the fact that, due to a remaining stock of Sumishield, 3 of our HLC households received Sumishield in Feb-March 2023 and 5 other HLC households received Actellic. Restricting our analysis to these 8 households, we used negative binomial regression to model overall and *An. funestus*-specific aHBRs across all available observations (6 of the 8 had observations in Phase 1 as well) with HLC collections included as an offset and accounting for household level clustering. Co-variates included the month and year of the observation. A dummy variable represented whether the household received Sumishield versus Actellic in Feb-March 2023 and a binary variable representing control versus intervention was set at “0” before the date the household was confirmed to have received the different IRS formulations and “1” thereafter. The interaction term between the IRS formulation and the date it was received is the “difference-in-difference” and was interpreted as the causal effect of receiving Sumishield versus Actellic IRS. This approach assumes that, in the absence of a difference in IRS formulations, the trajectory of nightly landing rates would have followed a similar path across both groups. For all comparisons, marginal estimates and rate ratios are presented with 95% confidence intervals and a p-value < 0.05 was considered statistically significant.

## Data Availability

The datasets used and/or analyzed during the current study are not yet publicly available but can be obtained from the corresponding author on reasonable request.
